# A *bifurcated palea* mutant infers functional differentiation of *WOX3* genes in flower and leaf morphogenesis of barley

**DOI:** 10.1093/aobpla/plac019

**Published:** 2022-05-05

**Authors:** Takanori Yoshikawa, Hiroshi Hisano, Ken-Ichiro Hibara, Jilu Nie, Yuki Tanaka, Jun-Ichi Itoh, Shin Taketa

**Affiliations:** Graduate School of Agriculture, Kyoto University, Kyoto 606-8502, Japan; Graduate School of Agricultural Regional Vitalization, Kibi International University, Minamiawaji, Hyogo 656-0484, Japan; Institute of Plant Science and Resources, Okayama University, Kurashiki, Okayama 710-0046, Japan; Graduate School of Agricultural Regional Vitalization, Kibi International University, Minamiawaji, Hyogo 656-0484, Japan; Graduate School of Agricultural and Life Sciences, University of Tokyo, Tokyo 113-8657, Japan; Graduate School of Agriculture, Kyoto University, Kyoto 606-8502, Japan; Graduate School of Agricultural Regional Vitalization, Kibi International University, Minamiawaji, Hyogo 656-0484, Japan; Graduate School of Agricultural and Life Sciences, University of Tokyo, Tokyo 113-8657, Japan; Institute of Plant Science and Resources, Okayama University, Kurashiki, Okayama 710-0046, Japan

**Keywords:** Barley, floral organ, *Hordeum vulgare*, leaf, trichome, WUSCHEL-RELATED HOMEOBOX 3

## Abstract

Barley (*Hordeum vulgare*) is the fourth most highly produced cereal in the world after wheat, rice and maize and is mainly utilized as malts and for animal feed. Barley, a model crop of the tribe Triticeae, is important in comparative analyses of Poaceae. However, molecular understanding about the developmental processes is limited in barley. Our previous work characterized one of two WUSCHEL-RELATED HOMEOBOX 3 (*WOX3*) genes present in the barley genome: *NARROW LEAFED DWARF1* (*NLD1*). We demonstrated that *NLD1* plays a pivotal role in the development of lateral organs. In the present study, we describe a *bifurcated palea* (*bip*) mutant of barley focusing on flower and leaf phenotypes. The palea in the *bip* mutant was split into two and develop towards inside the lemma surrounding the carpels and anthers. The *bip* mutant is devoid of lodicules, which develop in a pair at the base of the stamen within the lemma in normal barley. *bip* also exhibited malformations in leaves, such as narrow leaf due to underdeveloped leaf-blade width, and reduced trichome density. Map-based cloning and expression analysis indicated that *BIP* is identical to another barley *WOX3* gene, named *HvWOX3.* The *bip nld1* double mutant presented a more severe reduction in leaf-blade width and number of trichomes. By comparing the phenotypes and gene expression patterns of various *WOX3* mutants, we concluded that leaf bilateral outgrowth and trichome development are promoted by both *NLD1* and *HvWOX3*, but that *HvWOX3* serves unique and pivotal functions in barley development that differ from those of *NLD1*.

## Teaser

In the present study, we describe a *bifurcated palea*(*bip*) mutant of barley focusing on flower and leaf phenotypes. *bip*exhibited a variety of abnormal development in floral organs characterized by the split palea phenotype. *bip*also exhibited malformations in leaves, such as narrow leaf and reduced trichome density. Map-based cloning and expression analysis indicated that *BIP*is identical to one of the two barley *WOX3*genes, named *HvWOX3*. By comparing the phenotypes and gene expression patterns of various *WOX3*mutants, this study has highlighted both the functional conservation and differentiation of *WOX3*genes in barley.

## Introduction

Poaceae is one of the largest *Angiosperm* families and it includes many important agricultural crops such as wheat (*Triticum aestivum*), rice (*Oryza sativa*), maize (*Zea mays*) and barley (*Hordeum vulgare*). The inflorescence of Poaceae is composed of spikelets, and each spikelet contains one or more florets. The florets are initiated on an axis known as rachilla, and the number of florets per spikelet varies among species (Sakuma *et al.* 2011). In the case of barley, the rachilla phytomer degenerates after producing one floret, while it continues to develop and distichously articulates up to 12 florets in wheat (Forster *et al.* 2007). The floral organs (the lodicule, stamen and carpel) are surrounded by two grass-specific organs called the lemma and palea. Both organs are considered to represent reduced vegetative leaves, and the upper part of the lemma forms a long distal appendage, called the awn. The *leafy-lemma* (*lel*) mutant converts the lemma and awn into a leaf-like structure that is divided into distinct sheath and blade segments (Pozzi *et al.* 2000). The transition zone between lemma and awn of *lel* is marked by a fringe similar to a rudimentary ligule. Therefore, there are phytomeric similarities between lemma (lemma and awn) and leaf structure (sheath and blade). On the other hand, the palea is a bifid structure which protect floral organs along with the lemma (von Bothmer and Jacobsen 1985). A split palea mutant of barley suggested that the normal palea in barley represents two paired structures that are fused together at a common edge. Split and divided paleas are well known in other members of the Triticeae; therefore, the palea can be considered to have a similar origin as other paired structures of the plant such as coleoptile and lodicules (Forster *et al.* 2007).

A split palea phenotype is also reported in the rice *leaf lateral symmetry1* (*lsy1*) mutant (Honda *et al.* 2018). *LSY1* encodes WUSCHEL-related homeobox 3 (WOX3), and genes of *WOX3* play essential roles in the development of lateral domains in both vegetative and reproductive lateral organs. The first *WOX3* gene to be isolated was *PRESSED FLOWER* (*PRS*) in *Arabidopsis*, and mutations in *PRS* caused repressed growth of the lateral sepals and defects in cell files at the lateral margins (Matsumoto and Okada 2001). *PRS* transcripts were found to be localized in the lateral regions of flower primordia, floral organ primordia and young leaf primordia; however, no distinct defects in the leaf lateral regions were detected in *prs* mutants, with the exception of lacking stipules (Matsumoto and Okada 2001; Nardmann *et al.* 2004). An additional mutation in *WOX1* caused significant narrowing of the leaves in *prs* mutants. The *Arabidopsis WOX1* gene and its orthologous genes in petunia (*MAEWEST*), tobacco (*LAMINA1*) and *Medicago truncatula* (*STENOFOLIA*) belong to the same *WOX* family clade as *PRS* and are expressed in the marginal regions of the leaf and floral organ primordia (McHale 1992; Haecker *et al.* 2004; Vandenbussche *et al.* 2009; Tadege *et al.* 2011). *prs wox1* double mutants presented a loss of leaf marginal tissues and disordered adaxial–abaxial identities in the leaf marginal regions, suggesting that WOX members are involved in the downregulation of both adaxial and abaxial regulators at the adaxial–abaxial boundary (Nakata *et al.* 2012; Nakata and Okada 2013). *WOX1* appears to be absent from monocot families; however, several *WOX3* genes are present within monocot genomes, which can be further classified into two subclades (Zhang *et al.* 2010; **see****Supporting Information—Fig. S1**). One subclade contains maize *NARROW SHEATH1* (*NS1*) and *NS2*, and loss-of-function mutations in both *NS* genes result in absent marginal regions in leaves and floral organs (Scanlon *et al.* 1996; Scanlon and Freeling 1998; Nardmann *et al.* 2004). *NS* transcripts accumulate in the pre-marginal regions of leaf primordia and play critical roles in the recruitment of leaf founder cells by downregulating KNOX accumulation (Scanlon and Freeling 1997; Scanlon 2000; Scanlon *et al.* 2000). Similar developmental defects in lateral domains were observed in *NS* orthologue mutants in rice (*NARROW LEAF2* [*NAL2*], *NAL3*; Cho *et al.* 2013; Ishiwata *et al.* 2013) and barley (*NARROW LEAFED DWARF1* [*NLD1*]; Yoshikawa *et al.* 2016).

A variety of mutants in the *NS*-related subclade have been identified and characterized; however, little is known about the other *WOX3* subclade. The rice *OsWOX3* gene was the first to be identified from this subclade and was found to be the causal gene for the glabrous phenotypes of rice Acc IRGC104038 (*depilous*; Angeles-Shim *et al.* 2012), Jia64 (*Glabrous Rice 1*; Li *et al.* 2012) and HMK (*NUDA/GL-1*; Zhang *et al.* 2012). Recently, the *Oswox3* mutant, *lsy1*, was identified, which showed asymmetrical leaf defects and malformation in floral organs, as well as a lack of bristle-type trichomes (Honda *et al.* 2018). Notably, loss of function or overexpression of *LSY1* affected the localization of adaxial–abaxial regulators in leaf primordia, suggesting that *LSY1* may indirectly promote the expression of adaxial–abaxial regulators. The differences between the gene expression patterns and mutant phenotypes of *NAL2/3* and *LSY1* indicate functional diversification of *WOX3* in rice.

Despite its importance as the fourth major crop, knowledge regarding the developmental processes of barley is limited. Our previous study revealed that the barley genome contains two *WOX3* genes, *NLD1/HvNS* and *HvWOX3* (Yoshikawa *et al.* 2016; **see****Supporting Information—Fig. S1**). *nld1* mutants exhibit a clear reduction in the width of leaves, lemmas and paleae as they lack marginal regions. The expression patterns and phenotypes of *nld1* mutants are homologous to those of *NS1* and *NS2* in maize and *NAL2* and *NAL3* in rice, suggesting that their functions are conserved among *NS*-related genes in the development of lateral organs. In the present study, we identified a novel barley mutant, *bifurcated palea* (*bip*), named from the obvious phenotype of palea. *bip* exhibited various clear malformation in the reproductive organs such as the disconnection of the palea in the centre and absence of lodicules. *bip* also showed several abnormalities in the vegetative organs, and map-based cloning and expression analysis indicated that *BIP* is identical to *HvWOX3*.

## Materials and Methods

### Plant materials and growing conditions

*bip* is a gamma-ray-induced mutant derived from the barley line Kanto Nijo 29 (KN29), as is the same for *nld1.b* in a previous report (Yoshikawa *et al.* 2016). *bip* was identified by phenotypic observation for its irregular spike shape and semi-naked seed morphology. For the evaluation of mutant phenotypes, mutant and wild-type seeds were sown on soil and grown under natural conditions. For uniform germination, seeds were placed on wet paper at 15 °C for 3 days and then transplanted to soil.

### Map-based cloning of *BIP*

The genetic mapping population comprised 97 F_2_ plants derived from a cross between the *bip* mutant and normal barley (OUI026). For mapping, DNA was isolated according to Taketa *et al.* (2012). Public EST (Sato *et al.* 2009) markers were used for mapping. Genetic mapping was conducted according to Yuo *et al.* (2012). New DNA markers for fine mapping were developed as described by Taketa *et al.* (2011). The target chromosomal region generally showed a micro-synteny to a region on rice chromosome 5, but several markers corresponded to rice chromosomes 7, 8, 10 and 11. In total 12 polymorphic markers were used, and three of them were dominant markers. Primer sequences and the detection methods of polymorphisms are summarized in **Supporting Information—Table S1**. Critical recombinant plants for *bip* were subjected to F_3_ progeny tests. To know physical distances between barley genes, we also used the version of barley genome assembly Hv_IBSC_PGSB_v2 of cv. Morex (Mascher *et al.* 2017), as viewed from the Ensembl Plants *Hordeum vulgare* database (http://plants.ensembl.org/Hordeum_vulgare/Info/Index).

### Epidermal cell observation

The second leaf blades of the mutants and wild type were fixed using formaldehyde/glacial acetic acid/50 % ethanol (2:1:17) for 24 h at 4 °C. The blades were then dehydrated in a graded ethanol series. Dehydrated samples were incubated at 96 °C in chloralhydrate dissolved in 100 % ethanol until they were cleared, after which they were observed under a light microscope. Cell width was measured by image analysis using Image J (available at http://rsbweb.nih.gov/ij/).

### Paraffin sectioning and histological analysis

Mutant and wild-type plant samples were fixed using formaldehyde/glacial acetic acid/50 % ethanol (2:1:17) for 24 h at 4 °C for histological analysis, or fixed using 4 % (w/v) paraformaldehyde and 1 % Triton X in 0.1 M sodium phosphate buffer for 48 h at 4 °C for *in situ* hybridization. The samples were then dehydrated in a graded ethanol series, substituted with 1-butanol and embedded in Paraplast Plus (McCormick Scientific, LLC). The samples were sectioned to a thickness of 8 μm using a rotary microtome (HM340E, MICROM). For the histological analyses, sections were stained in haematoxylin. After staining, sections were mounted with Poly-Mount (Polysciences, Inc.) and observed under a light microscope.

### *In situ* hybridization

Paraffin sections were prepared as mentioned above. Digoxigenin-labelled anti-sense and sense RNA probes were prepared using a 736-bp cDNA template of *HvWOX3*, which was amplified by PCR using a forward primer (5ʹ-ATCCTGGAGGAGATGTACCG-3ʹ) and a reverse primer (5ʹ-GCTGCTCCTCCTTGATCG-3ʹ), as well as a 666-bp cDNA template of *NLD1*, which was amplified using a forward primer (5ʹ-AGCAGCTGATGATCCTGGAG-3ʹ) and a reverse primer (5ʹ-AGGTGGAGCAAGAGGAGGAC-3ʹ). Amplified PCR products were cloned into the pCR-Blunt vector (Invitrogen), followed by *in vitro* transcription using the DIG RNA Labelling Kit (Roche, Ltd.). *In situ* hybridization and immunological detection using alkaline phosphatase were performed as described previously (Kouchi and Hata 1993). For double-target *in situ* hybridization, digoxigenin-labelled and biotin-labelled probes were used. Probe hybridization, post-hybridization washes and blocking procedures were performed using previously described methods (Kouchi and Hata 1993). The TSA Biotin System (PerkinElmer, Inc.) and HNPP Fluorescent Detection Set (Roche) were used for the detection of biotin-labelled and digoxigenin-labelled probes, respectively, according to the manufacturer’s instructions. The slides were washed in sterile distilled water, mounted using Prolong Gold Antifade Reagent with DAPI (Invitrogen) and then observed under a fluorescence microscope.

## Results

### Reproductive phenotypes of the *bip* mutant

**Figure 1.**
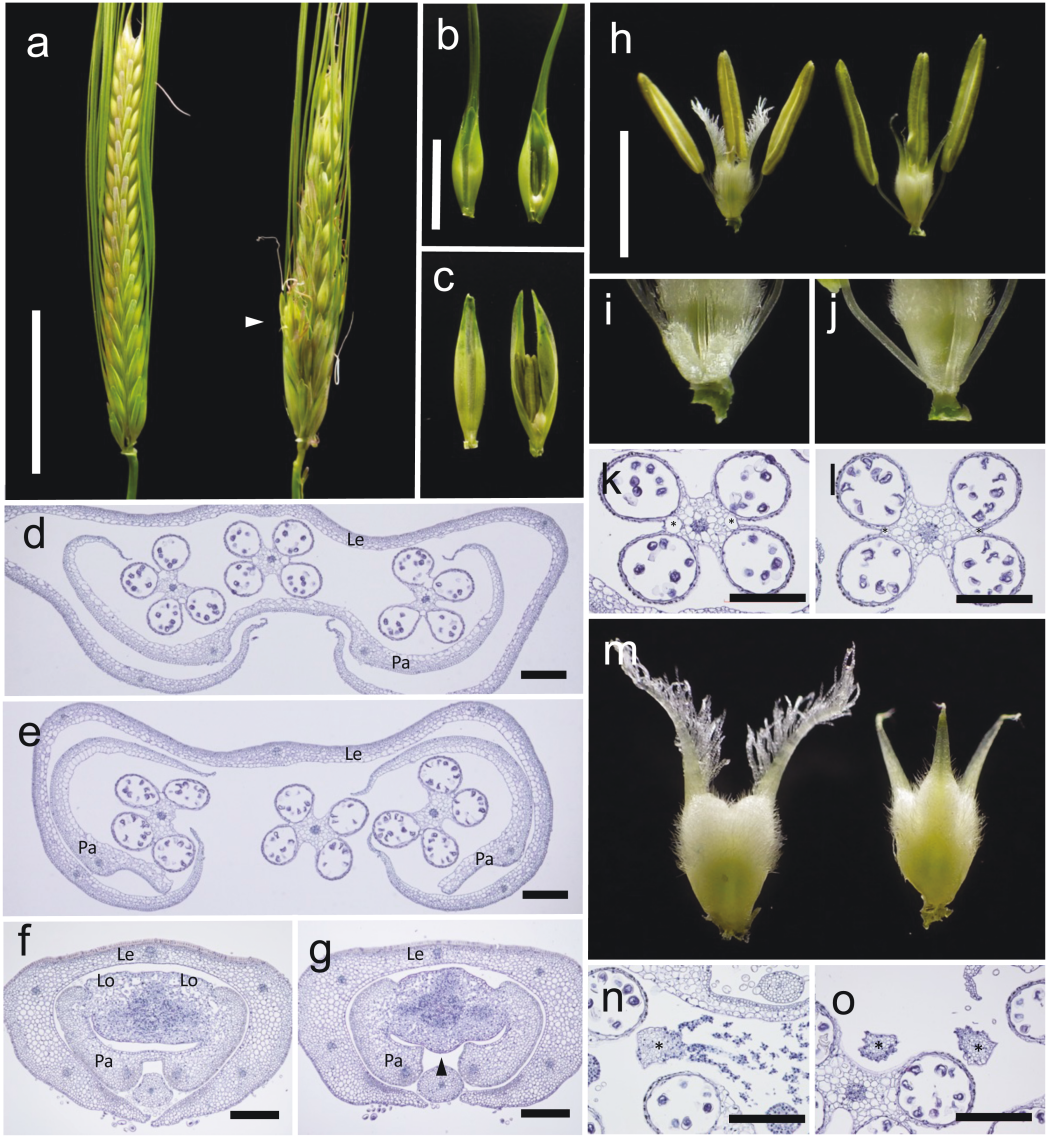
Reproductive phenotypes of wild type and *bip*. (A–C) The spikes (A) and spikelets (B, C) of wild type (left) and *bip* (right). The arrow head in (A) indicates ectopic branching in *bip* spike. The lemmas are removed in (C). (D–G) The cross-sections of spikelets at the middle part (D, E) and the basal part (F, G) in wild type (D, F) and *bip* (E, G). The arrow head in (G) indicates disconnected palea in the mutant. Le, lemma; Pa, palea; Lo, lodicule. (H–J) The reproductive organs of wild type (left) and *bip* (right). The lodicules in (H) are enlarged in (I) (wild type) and (J) (*bip*). (K, L) The cross-sections of anther in wild type (K) and *bip* (L). The asterisks indicate dehiscence cavities. (M) The carpels of wild type (left) and *bip* (right). (N, O) The cross-sections of stigmata in wild type (N) and *bip* (O). The asterisks indicate stigmata. Scale bars = 3 cm (A), 1 cm (B), 200 μm (D–G, K, L, N, O) and 5 mm (H).

The *bip* mutant exhibited a variety of abnormal phenotypes in the reproductive organs. The arrangement of spikelets was disordered due to ectopic branching in *bip* spikes (Fig. 1A). While clear malformations were not observed in the lemma, the palea was split in the centre, which resulted in exposure of the reproductive organs (Fig. 1B–E). The lodicules were mostly absent in the mutant (Fig. 1F–J). The dehiscence cavities were poorly developed in *bip* anthers (Fig. 1K and L), and the stigmata mostly lacked branches in the mutant strain (Fig. 1M–O); these features are likely the cause of the relatively low seed setting rate observed in the mutant. The stigmata were frequently trifurcated (Fig. 1M), and the ovules were exposed in the bifurcated carpels in *bip***[see****Supporting Information—Fig. S2****]**. These results suggested that *BIP* is essential for the proper development of reproductive organs.

### Vegetative phenotypes of the *bip* mutant

**Figure 2.**
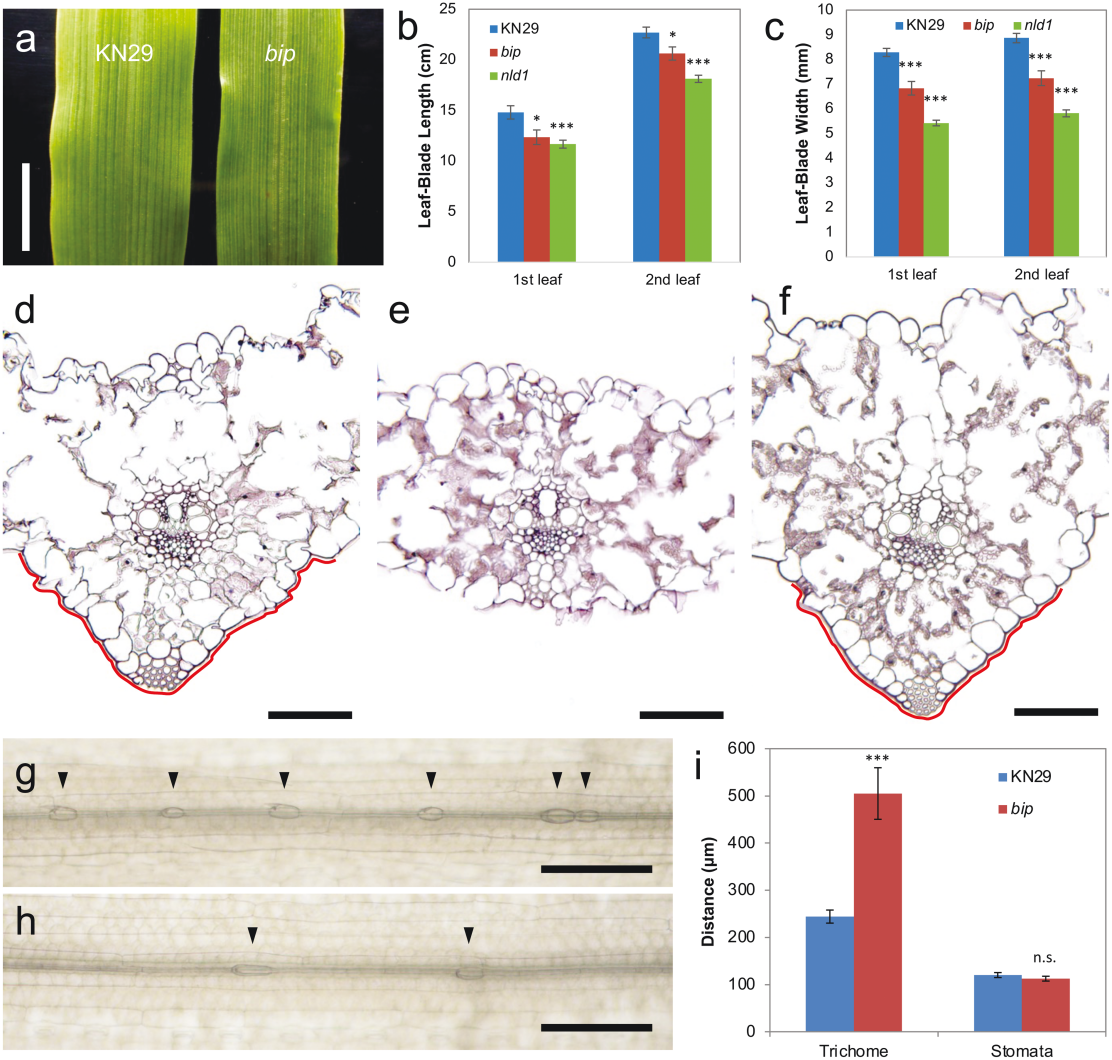
Leaf-blade phenotypes of wild type, *bip* and *nld1* mutants. (A) Leaf blades of the second leaf in wild type and *bip*. (B, C) Comparison of leaf-blade length (B) and width (C) among wild type, *bip* and *nld1.b*. (D–F) The cross-sections of the central part of the second leaf blade in wild type (D), *bip* (E) and *nld1.b* (F). The protrusion on the abaxial side of the leaf blade is highlighted with bold line in (D) and (F). (G, H) The distribution of trichomes on the adaxial side of the second leaf blades in wild type (G) and *bip* (H). Arrow heads indicate the positions of trichomes. (I) Comparison of trichome and stomata distances in the second leaves between wild type and *bip*. Results are shown as mean ± SE (*n* = 10) (B, C, F). Asterisks indicate results that were significantly different from the wild type at *P < 0.05, **P < 0.01 and ***P < 0.001 (*t*-test). Scale bars = 5 mm (A), 100 μm (D–F), 200 μm (G, H).

In addition to the reproductive phenotypes, *bip* exhibited several abnormalities in vegetative organs. The leaf-blade length and width were decreased compared with wild type (Fig. 2A–C). The protrusion on the abaxial side of the leaf-blade centre was lacked in *bip* (Fig. 2D and E). Additionally, *bip* had longer intervals between trichomes on the leaf-blade surface than did the wild type, although the intervals between stomata were comparable (Fig. 2G–I).

### Map-based cloning of *BIP*

**Figure 3.**
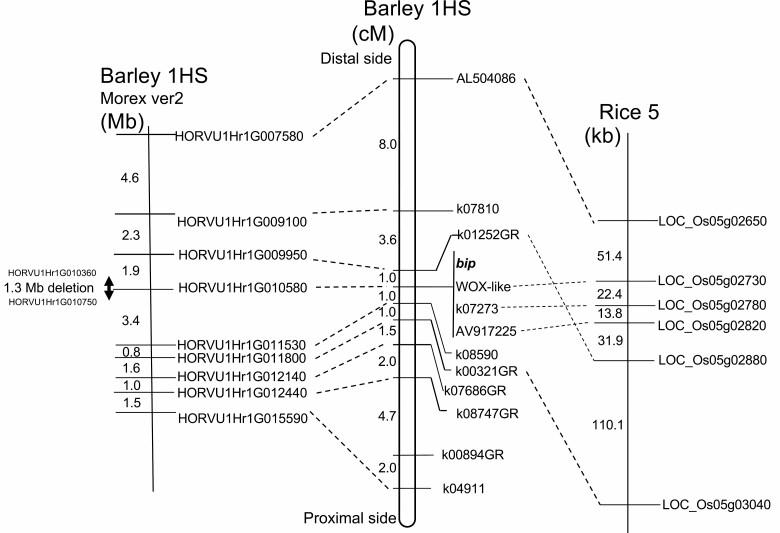
Fine mapping of the *bip* locus. Left is a physical map of barley chromosome 1H. Middle is a genetic map around the *bip* locus. Three markers co-segregating with *bip* are shown besides a vertical bar. Right is a physical map of the syntenic region of rice chromosome 5. Homologous markers are connected through dotted lines. Barley markers without connection are homologous to rice chromosomes other than 5.

Genetic mapping was conducted using 97 F_2_ plants derived from a cross between *bip* and normal barley (OUI026). The *bip* locus was localized within a 2.0-cM interval flanked by markers k01252GR and k08590, respectively, at the 1.0-cM both distal and proximal sides in the proximal region of the barley chromosome arm 1HS (Fig. 3). Then, we exploited micro-colinearity of our barley genetic map to the rice reference genome sequence of cv. Nipponbare using the MSU Rice Genome Annotation Project Database (http://rice.plantbiology.msu.edu/). The gene order in this barley region is rather conserved with a syntenic region of rice chromosome 5, but one flanking marker k01252GR twisted on rice chromosome 5. However, three dominant markers (WOX-like, k07273 and AV917225) co-segregated with *bip*, and their rice putative orthologues resided on a near rice 5 chromosome region. These three genes are considered candidates of the *BIP* locus. Among others, WOX-1 like (CAJX010219123) is the most plausible candidate gene for *BIP*. This is because loss of function of *OsWOX3/LSY1* (LOC_Os05g02730) causes asymmetrical leaf defects, lack of bristle-type trichomes, separated palea and exposure of ovule (Honda *et al.* 2018). Similarly, *bip* also showed separated palea, exposure of ovule, reduced leaf size and decrease of trichome density, and *HvWOX3* (*HORVU1Hr1G010580*) gene is located within the barley candidate region. Therefore, we attempted to compare the nucleotide sequence of *HvWOX3* between wild type (KN29) and *bip*; however, the PCR product of *HvWOX3* was not obtained only from *bip***[see****Supporting Information—Fig. S3****]**. Further experiments to amplify the neighbouring genes indicated that about 1.3-Mb region containing *HvWOX3* was lost in *bip* (Fig. 3; **see****Supporting Information—Table S2**); the primer pairs designed for genes between *HORVU1Hr1G010360* and *HORVU1Hr1G010750* did not amplify the expected amplicons from *bip*, but the same primer pairs amplified from wild type. It was, therefore, considered that the *bip* mutant has a chromosomal deletion of about 1.3 Mb in size caused by gamma-ray irradiation, and revealed that *bip* is a *Hvwox3*-null mutant.

**Figure 4.**
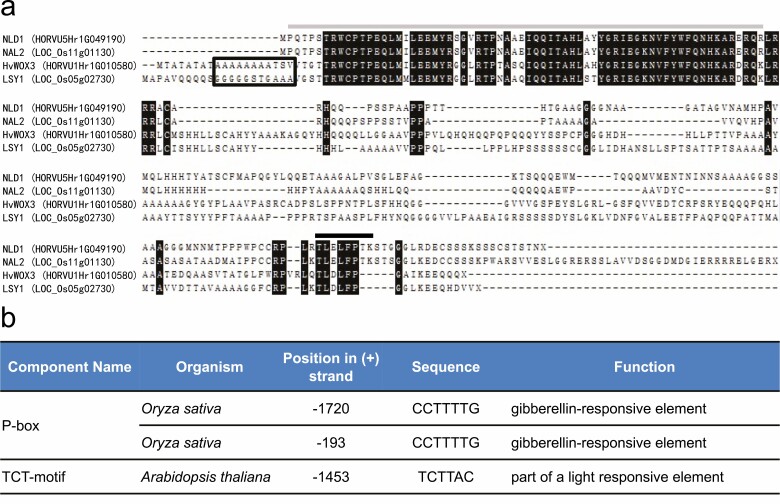
Analysis of the amino acid sequences and nucleotide sequences of promoter regions in *HvWOX3*-related genes. (A) Comparison of the amino acid sequences of NLD1 (HORVU5Hr1G049190), NAL2 (LOC_Os11g01130), HvWOX3 (HORVU1Hr1G010580) and LSY1 (LOC_Os05g02730). Amino acid sites with 100 % similarity following sequence alignment are coloured in black. The grey line indicates the homeobox domain. The black line indicates the WUS-box motif. The black box contains a cluster of acidic amino acids. (B) The putative *cis*-acting regulatory elements common to both the *HvWOX3* and *LSY1* promoters. Located positions upstream from the start codon of *HvWOX3* in barley genome sequence are indicated.

*HvWOX3* contains an open reading frame of 846 bp that encodes a protein of 281 amino acids with an estimated molecular mass of 30.44 kDa and a calculated protein isoelectric point of 8.73 (Fig. 4A). Sequence alignment was performed to compare the protein and promoter sequences of *HvWOX3* with those of three related genes: barley *NLD1* (*HORVU5Hr1G049190*), rice *NAL2* (*LOC_Os11g01130*) and rice *LSY1***[see****Supporting Information—Fig. S1****]**. HvWOX3 shared 43.96 % identity with NLD1, 48.54 % identity with NAL2 and 56.11 % identity with LSY1 (Fig. 4A). Each of the genes examined possessed high levels of sequence similarity in the Homeobox domain regions (amino acids 2–67 in NLD1 and NAL2, 29–84 in HvWOX3 and 21–86 in LSY1). For each of the aligned sequences, 51 amino acids were 100 % conserved in the Homeobox domain regions; each sequence contained the distinctive WUSCHEL-box motif T-L-X-L-F-P-X-X, where X is any amino acid in the carboxyl terminal (Haecker *et al.* 2004; Fig. 4A). A cluster of acidic amino acids upstream of the Homeobox domain that is common to both the *HvWOX3* and *LSY1* sequences could potentially act as an activator domain. To explore the potential functions of *HvWOX3*, the *cis*-acting elements in the promoter regions of the selected genes were predicted using PlantCARE. The *cis*-acting elements that were commonly predicted within the *HvWOX3*, *NLD1*, *NAL2* and *LSY1* promoters include phytohormone response elements, light response elements and the binding sites of typical transcription factors **[see****Supporting Information—Table S3****]**. P-box and TCT motifs were present in the promoters of *HvWOX3* and *LSY1*, but not in *NLD1* or *NAL2* (Fig. 4B).

### Abnormal development of the *bip nld1* double mutant

**Figure 5.**
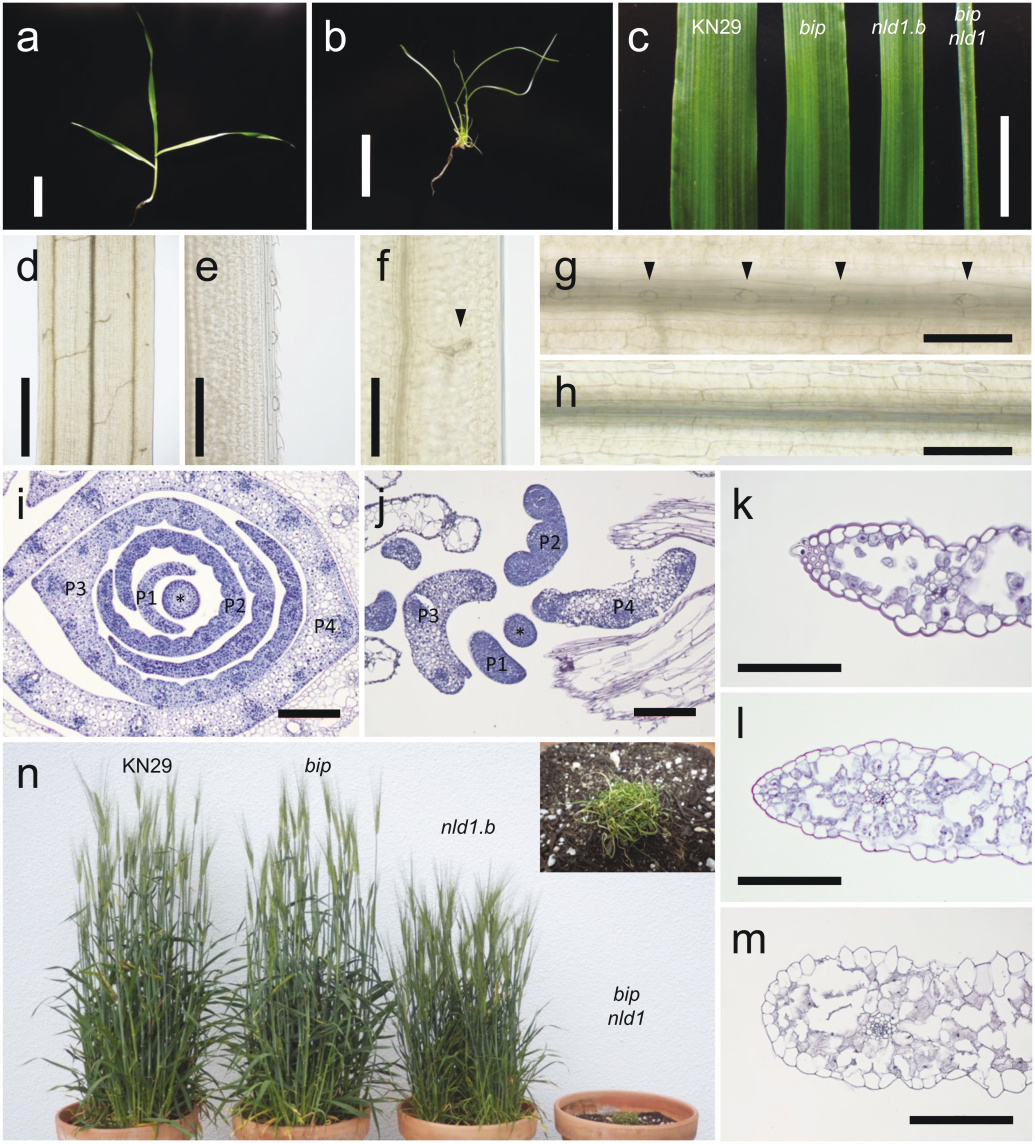
Vegetative phenotypes of wild type and *wox3* mutants. (A, B) Seedlings of wild type (A) and *bip nld1* double mutant (B). (C) Leaf blades of the second leaf in wild type, *bip*, *nld1.b* and double mutant. (D) A cleared leaf blade of the *bip nld1* double mutant. (E, F) Leaf margins of the second leaf blades in wild type (E) and *bip nld1* double mutant (F). The arrow head in (F) indicates abnormal commissural vein. (G, H) The distribution of trichomes on the adaxial side of the second leaf blades in wild type (G) and *bip nld1* double mutant (H). Arrow heads in (G) indicate the positions of trichomes. (I–M) Cross-sections of shoot apexes (I, J) and leaf margins of the second leaf blades (K–M) in wild type (I, K), *nld1.b* (L) and *bip nld1* double mutant (J, M). The asterisks and P1–P4 indicate SAMs and leaf primordial stages, respectively. (N) Matured plants of wild type, *bip*, *nld1.b* and double mutant. *bip nld1* double mutant is enlarged in the figure. Scale bars = 5 cm (A, B), 1 cm (C), 1 mm (D), 200 μm (E–M).

While *bip* showed a disconnection of palea in the centre and lack of protrusion on the abaxial side of the leaf blade, *nld1* exhibited lack of marginal regions in the palea and lemma (Yoshikawa *et al.* 2016), and there was no obvious alteration in the leaf-blade protrusion (Fig. 2F). On the other hand, *nld1* exhibited much severer reduction in the leaf size than *bip*, particularly in the width direction (Fig. 2B and C), due to the lack of marginal region, but there was no clear malformation in the marginal organs such as auricles and sawtooth hairs in *bip***[see****Supporting Information—Fig. S4****]**. These differences imply functional diversification of *NLD1* and *BIP* in barley development. However, *bip nld1* double mutants derived from the cross between *bip* and *nld1.b* exhibited more severe phenotypes than those of either single mutant. The plants were clearly dwarfed, and the leaf blades were much narrower than those of *nld1.b* (Fig. 5A–C). No more than three longitudinal veins were formed in the leaf blades, and commissural veins were frequently disconnected in the double mutant (Fig. 5D). No sawtooth hairs had developed in the leaf edges, and the commissural veins developed abnormally in the marginal regions (Fig. 5E and F); this was also reported in *nld1* mutants (Yoshikawa *et al.* 2016). Significantly fewer trichomes were present on the leaf-blade surface in the double mutant (Fig. 5G and H) compared with *bip* (Fig. 2G and H). The width of the leaf primordia increased with primordial stage in wild type and *nld1*; however, the growth rate of the leaves was significantly lower in the double mutant (Fig. 5I and J), suggesting that growth in the mediolateral direction could be suspended during primordial development. Cross-sections of the leaf margins revealed that sawtooth hair and sclerenchymatous cell development were defective, and that the thickness of leaf edges in the double mutant was increased compared with wild type or *nld1.b* (Fig. 5K–M). At the adult stage, the plant height of *bip* was comparable with that of the wild type, and *nld1.b* presented a dwarfed phenotype compared with the wild type, as reported previously (Yoshikawa *et al.* 2016; Fig. 5N). On the other hand, internode elongation was not observed in the double mutant, and the plants withered before reaching the reproductive phase. The phenotypes exhibited by the double mutant suggested that *NLD1* and *BIP* are both involved in leaf-blade outgrowth and trichome development with some degree of functional redundancy, although each gene appears to be different in contribution to plant development.

### Expression analysis of *WOX3* genes

**Figure 6.**
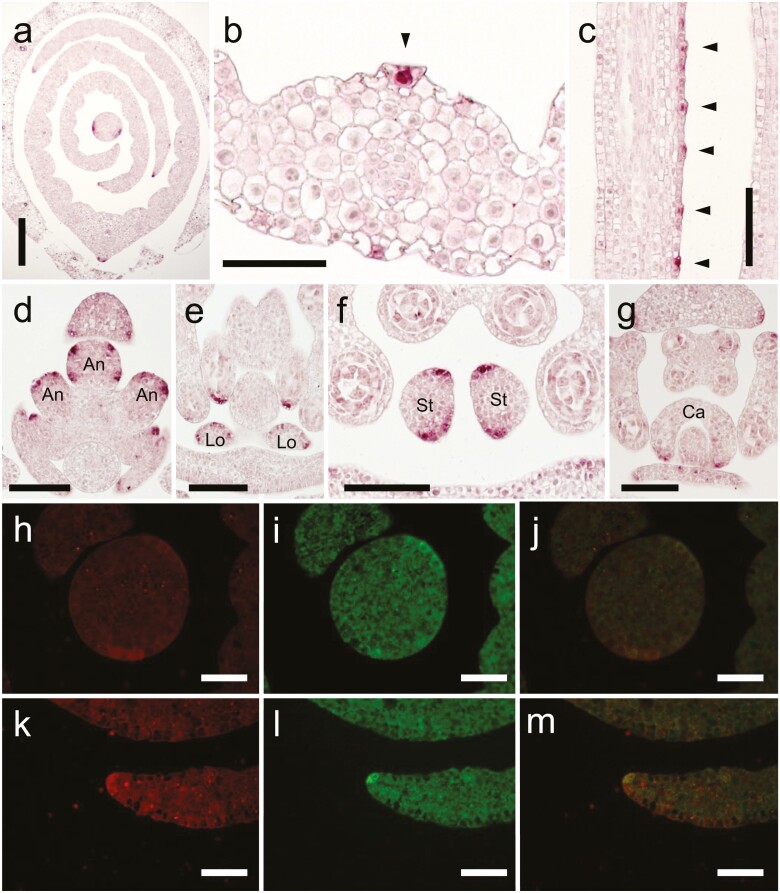
Expression patterns of the *HvWOX3* and *NLD1* genes in wild type. (A–G) Cross-sections of shoot apex at the second-leaf stage (A), leaf primordium (B), anthers (D), stigmata (F) and carpel (G) and longitudinal sections of leaf primordium (C) and lodicules (E) in wild type hybridized with *HvWOX3* anti-sense probe. Arrow heads in (B) and (C) indicate the positions of developing trichomes. An, Lo, St and Ca in the figures indicate anther, lodicule, stigma and carpel, respectively. **See****Supporting Information—Fig. S6** for sense probe. (H–M) Double-target *in situ* hybridization of *HvWOX3* and *NLD1* genes in the shoot apex of wild-type plants at the second-leaf stage. Cross-sections of SAM (H–J) and P2 leaf primordium (K–M) were hybridized with anti-sense probes for *HvWOX3* (H, K) and *NLD1* (I, L). Merged views of (H) with (I) and of (K) with (L) are shown in (J) and (M), respectively. Scale bars = 200 μm (A), 50 μm (B, H–M) and 100 μm (C–G).

To further investigate the function of *HwWOX3* in barley development, we examined the expression pattern of *HvWOX3* using *in situ* hybridization. During the process of leaf development, *HvWOX3* expression was initially observed in the pre-marginal regions of initiating leaf ridges in shoot apical meristem (SAM; Fig. 6A). In the developing leaf primordia, *HvWOX3* transcripts were localized at the leaf marginal edges, the abaxial surface of the central part and developing trichomes (Fig. 6A–C). In the reproductive organs, *HvWOX3* transcripts were localized at the marginal edges of paleae and lemmas, the corners of anthers, the surfaces of lodicules, the adaxial and abaxial surfaces of stigmata and the marginal edges of developing carpel (Fig. 6D–G). Many of these localizations corresponded to the phenotypic alterations in the *bip* mutant, supporting that *BIP* is identical to *HvWOX3*.

The marginal expression patterns of *HvWOX3* in the lateral organs were similar to those of *NLD1* (Yoshikawa *et al.* 2016). Double-target *in situ* hybridization showed that both transcripts were co-localized in the pre-marginal regions and marginal edges in SAMs and leaf primordia, respectively (Fig. 6H–M). These similarities in expression patterns, together with high nucleotide sequence homologies, could explain the functional redundancy between *HvWOX3* and *NLD1* in barley leaf development.

**Figure 7.**
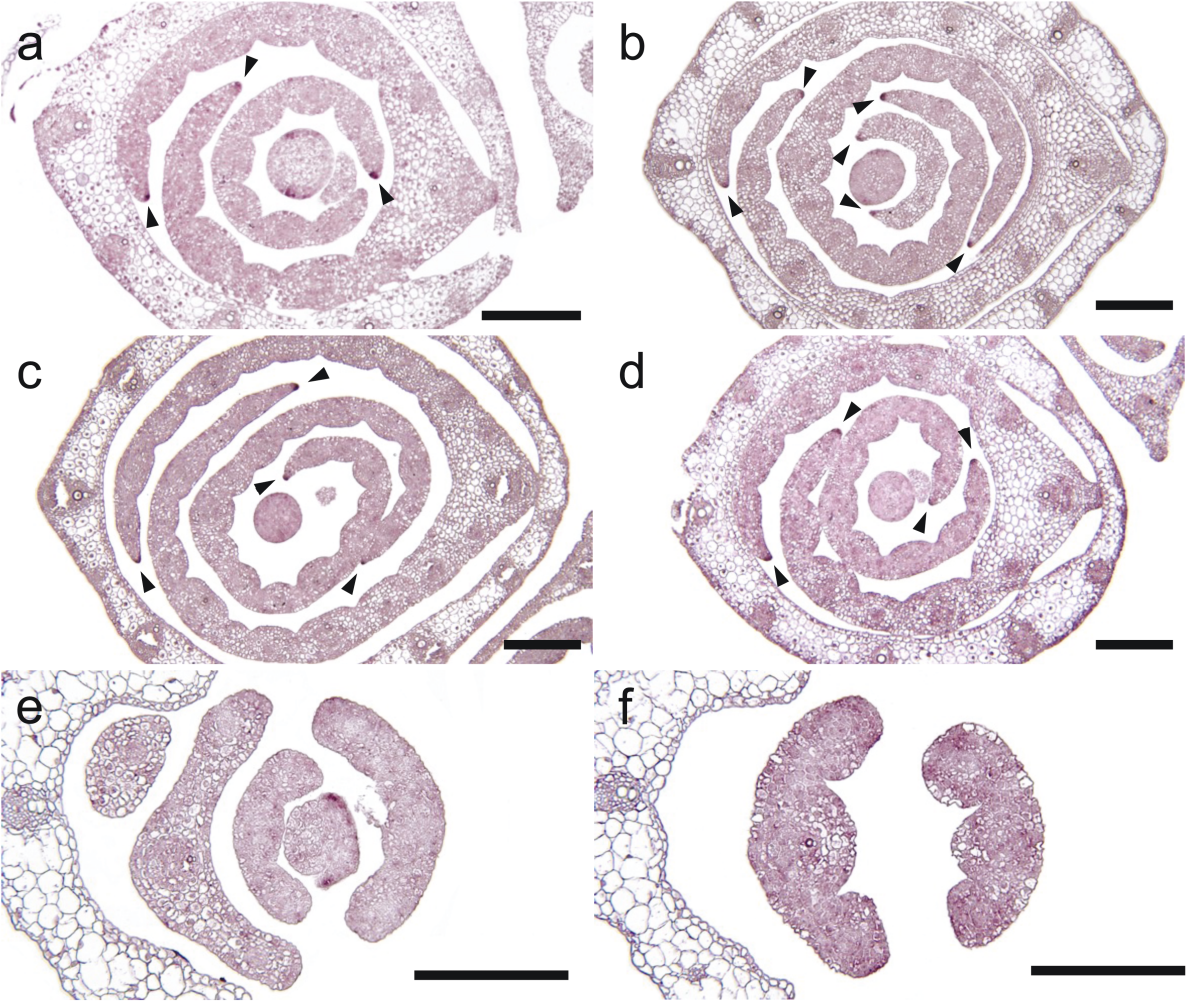
Expression patterns of the *HvWOX3* and *NLD1* genes in wild type and mutants. (A) A cross-section of the shoot apex at the second-leaf stage in *nld1* hybridized with a *HvWOX3* anti-sense probe. (B–F) Cross-sections of shoot apex at the second-leaf stage (B–E) and leaf primordia (F) in wild type (B), *bip* (C), *nld1.b* (D) and *bip nld1* double mutant (E, F) hybridized with a *NLD1* anti-sense probe. Arrow heads in (A–D) indicate localized signals at the leaf margins. Scale bar = 200 μm.

To examine the epistasis between *HvWOX3* and *NLD1* in gene expression regulation, their spatial expression patterns were investigated in the *bip* and *nld1* mutants. The expression pattern of *HvWOX3* in the *nld1* mutant was similar to that in wild type (Figs 6A and 7A). Likewise, the expression pattern of *NLD1* in *bip* also resembled that in wild type (Fig. 7B and C). These results indicated that the *WOX3* genes were expressed independently of each other. Although *nld1.b* lost function of the *NLD1* gene due to a 1-bp deletion in the first exon (Yoshikawa *et al.* 2016), the localization of *nld1.b* transcripts was comparable with that of *NLD1* transcripts in wild type (Fig. 7B and D). However, *nld1.b* transcripts were not distinctly localized in the marginal region of leaf primordia in the *bip nld1* double mutant (Fig. 7E and F). This suggested that normal function of at least one of the *WOX3* genes is essential for the localized expression of *WOX3* genes.

## Discussion

Previous studies have demonstrated that *WOX3* genes play pivotal roles in the development of lateral domains in lateral organs. In monocot families, a variety of *WOX3* mutants have been identified; several previous studies have investigated gene function and expression in the *NS*-related subclade, but little is known about the other *WOX3* subclade. The present study identified the *bip* mutant with a variety of malformation in the floral organs, and map-based cloning and expression analysis indicated that *BIP* is identical to *HvWOX3*. While the *nld1* mutant exhibited a clear reduction in leaf-blade width, *bip* harboured a less dramatic reduction in leaf-blade width (Fig. 2A–C). However, the *bip nld1* double mutant exhibited a greater reduction in leaf-blade size (Fig. 5A–C), and we demonstrated that this was not simply additive effects of two independent *WOX3* mutant genes. These phenotypic alterations, together with the homologous nucleotide sequence and localization of transcripts in the marginal regions (Figs 4A and 6K–M), strongly suggested that *NLD1* and *HvWOX3* exert a conserved function to increase leaf size, particularly the leaf width. As well as a reduction in leaf-blade width, *nld1* presented abnormal stem development and a lack of leaf marginal tissues, which were not observed in the *bip* mutant. In addition, *HvWOX3* transcripts were localized to several reproductive organs (Fig. 6D–F), but these expression patterns were not observed in *NLD1* (Yoshikawa *et al.* 2016). Comparison of *cis*-acting regulatory elements in the promoter sequences revealed that P-box and TCT motifs were present in *HvWOX3* and *LSY1* promoters, but not in *NLD1* and *NAL2* promoters (Fig. 4B). Thus, further study is required to determine whether these two *cis*-acting elements are responsible for the differences in expression patterns between *HvWOX3* and *NLD1*. By comparing mutant phenotypes and gene expression patterns, this study has highlighted both the functional conservation and differentiation of *WOX3* genes in barley. However, the above observations should be carefully interpreted because the *bip* mutant is caused by a 1.3-Mb chromosomal deletion including the *HvWOX3* gene plus additional genes inside. Part of abnormalities in the *bip* mutant may be caused by combined effects of the absence of other genes that were included in the deletion. To substantiate the present results, other mutant alleles of the *BIP* locus are highly anticipated.

*bip* exhibited the obvious malformation in palea, which was also observed in *lsy1* mutants (Honda *et al.* 2018). However, no abnormal development was observed in the lemma of *bip*. Although the lemma and palea are facing organs, they originate from different axes; the lemma originates from the main axis and the palea from the floret axis (Lombardo and Yoshida 2015). Lemmas and paleae are considered to be bracts and prophylls, respectively, and are often considered distinct organs. This idea is supported by discovery of the *leafy-lemma* mutant of barley, the lemma of which transformed into a well-formed but diminutive leaf without affecting palea development (Pozzi *et al.* 2000). In another study, a split palea mutant of barley was identified, suggesting that normal paleae represent two paired structures that are fused together at a common edge (Forster *et al.* 2007). Therefore, the split palea phenotype of *bip* may indicate that *HvWOX3* is involved in the development and fusion of the edges of the two palea structures.

*bip* also exhibited the obvious malformations in the reproductive organs such as poor development of the dehiscence cavities in the anther, lack of branches in the stigmata and trifurcated stigmata in the carpel (Fig. 1K–O). Malformations in the reproductive organs were also observed in *lsy1*. Stamens produce microsporangia (pollen sacs) and carpels produce megasporangia (ovule), which in turn produce pollen and pollen tubes with two sperm cells and embryo sac with an egg cell, respectively; it can be considered that they are both evolved by the modification of sporophylls (Coen 1991; Endress 2001). Abnormal developments in the stamen and carpel in *bip* and *lsy1* suggest that both *HvWOX3* and *LSY1* appear to be involved in the differentiation of microsporophyll and megasporophyll in barley and rice, respectively. While the ovules are completely enclosed by carpels in wild type, it was exposed in the bifurcated *bip* carpels **[see****Supporting Information—Fig. S2****]**, which was also reported in *lsy1*. In rice, the floral meristem is converted into an ovule primordium, which is subsequently enclosed by carpel completely, forming ovary locule (Itoh *et al.* 2005). The exposure of ovule in *bip* and *lsy1* and marginal expression of *HvWOX3* in the developing carpel (Fig. 6G) indicate the involvement of *WOX3* genes in carpel development. Interestingly, similar ovule exposure was also reported in the loss of function of *WOX1* genes in tobacco (*LAMINA1*), *M. truncatula* (*STENOFOLIA*) and, more recently, tomato (*SlWOX1*; McHale 1992; Tadege *et al.* 2011; Zhang *et al.* 2020), while it was not observed in *prs* mutant. In this sense, *HvWOX3* and *LSY1* play more closer role in the development of carpel to *WOX1* than to *PRS*. Since *WOX1* appears to be absent from monocot families, it was speculated that a part of *WOX1* function is performed by *WOX3* genes in monocot families.

Localized expression in the marginal regions of lateral organs is a unique feature common to the *WOX3* genes. Even in the *WOX3* loss-of-function mutants, this distinct expression pattern persisted (Fig. 7A–D). However, *nld1.b* transcripts were not clearly localized to the marginal region of leaf primordia in the *bip nld1* double mutant (Fig. 7E and F). Leaf margins are established at the juxtaposition between the adaxial and abaxial cell populations, where they function as adaxial–abaxial boundaries; here, adaxial and abaxial regulators are downregulated by *WOX* genes (Nakada and Okada 2013). The loss of both *NLD1* and *HvWOX3* functions likely resulted in dysregulation of adaxial–abaxial regulators in the marginal region, thus causing delocalized expression of *WOX3* in the double mutant.

Rice *OsWOX3* was found to be essential for bristle-type trichome formation on the surface of leaves and glumes (Angeles-Shim *et al.* 2012; Li *et al.* 2012; Zhang *et al.* 2012). The recently identified *lsy1* mutant harboured asymmetrical leaf defects and malformation in floral organs, as well as a lack of bristle-type trichomes (Honda *et al.* 2018). In the present study, *bip* also displayed an obvious reduction in trichome density, suggesting that trichome formation is a conserved function of *WOX3* genes between rice and barley. While the bristle-type trichomes were completely absent in *lsy1*, a lack of trichomes on leaf surfaces was observed in the *bip nld1* double mutant only. A previous study reported that *NLD1* was expressed in immature trichome cells and that the *nld1* mutant showed slight abnormalities in trichome development (Yoshikawa *et al.* 2016). These results strongly suggest that the trichome formation function is conserved in *HvWOX3* and *NLD1* in barley, whereas the bristle-type trichome development is controlled by *LSY1* in rice. In addition, asymmetrical leaf defects, reported in *lsy1*, were not observed in *bip* but were occasionally present in *nld1*. Thus, we hypothesize that some rice *LSY1* functions, such as trichome formation and symmetrical leaf development, are performed by *NLD1* in barley. These differences between rice and barley could be explained by variations in the developmental mechanisms between rice bristle-type trichomes and barley trichomes, or the evolutionary loss of trichome formation function in *NAL2* and *NAL3* in rice. In either case, trichome formation appears to be the ancestral function of *WOX3* genes, as well as leaf outgrowth. Given that *NLD1* and *HvWOX3* retain both trichome formation and leaf outgrowth functions, barley could be a useful model to further investigate the expression, regulation and domain functions of WOX3 proteins.

Although the development of the protrusion on the leaf blade was comparable with that of wild type in *nld1*, it was not present in *bip* (Fig. 2D–F). Furthermore, *HvWOX3* transcripts were localized on the abaxial surface of the central part of leaf primordia as well as in the marginal regions (Fig. 6A); however, localization in the leaf central part was not observed in *NLD1***[see****Supporting Information—Fig. S5****]**. These results strongly suggest that *HvWOX3* is involved in the development of the protrusion in barley leaves. While many *Angiosperm* species form flattened bifacial leaves by developing in bilateral directions, several monocot species develop abaxialized unifacial leaves, in which the leaf blades extend in the dorsoventral direction. A previous study in *Juncus* species (commonly known as rushes) showed that the expression of *PRSb*, which belongs to the same subclade as *HvWOX3***[see****Supporting Information—Fig. S1****]**, was detectable in the margin-like regions of flattened leaf blades of *Juncus prismatocarpus.* However, *PRSb* was not detected in the cylindrical leaf blades of *Juncus wallichianus*, suggesting that *PRSb* may also regulate the flattening of unifacial leaf blades by promoting marginal growth (Yamaguchi *et al.* 2010). Since one of the margin-like regions of *J. prismatocarpus* leaves is equivalent to the abaxial surface of the central part of bifacial leaves, the expression pattern of *HvWOX3* is likely homologous to that of *PRSb* in developing leaf primordia. Taken together, these data suggest that genes of the *HvWOX3* subclade can participate in the outgrowth of leaf blades in multiple directions; this is a novel finding for *WOX3* genes.

In this study, we identified a *HvWOX3-null* mutant and investigated its phenotypic malformations. By comparing the phenotypic differences among the mutants *bip*, *nld1* and *bip nld1* double mutant, we discovered that leaf bilateral outgrowth and trichome development were promoted by both *NLD1* and *HvWOX3*, although to differing degrees. We also found that *HvWOX3* and rice *LSY1* play pivotal roles in floral organ development. Furthermore, we showed that *HvWOX3*, similar to *Juncus PRSb*, is involved in non-bilateral leaf outgrowth. These results suggested that the *HvWOX3* subclade likely obtained its unique functions throughout the course of evolution. The present study has revealed novel roles of the transcription factor *HvWOX3* in barley, which will aid future investigations into barley development.

## Supporting Information

The following additional information is available in the online version of this article—

Excel file: raw data

**Table S1.** PCR primers used for genetic mapping of *BIP*.

**Table S2.** Chromosomal deletion analysis surrounding the *BIP* locus using primers that were designed on the bases of the version of barley genome assembly Hv_IBSC_PGSB_v2 of cv. Morex.

**Table S3.** The putative *cis*-acting regulatory elements commonly included in the promoter of *WOX3* genes in barley and rice.

**Figure S1.** Phylogenetic tree of WOX3-related proteins. WOX3-related proteins in barley (NLD1/HvNS; HORVU5Hr1G049190, HvWOX3; HORVU1Hr1G010580), maize (NS1; NP_001105160, NS2; NP_001105242, ZmWOX3A; NP_001106239, ZmWOX3B; NP_001106240), rice (NAL2; LOC_Os11g01130, NAL3; LOC_Os12g01120, DEP/LSY1/OsWOX3; LOC_Os05g02730), sorghum (SbNS; XP_002449019, SbWOX3; XP_002440499), *Brachypodium distachyon* (BdNS; XP_010238840, BdWOX3; XP_003566641), *Juncus prismatocarpus* (Jp PRSa; AB539880, Jp PRSb; AB539881), *Juncus wallichianus* (Jw PRSa; AB539883, Jw PRSb; AB539884) and *Arabidopsis thaliana* (PRS; AT2G28610, AtWOX1; AT3G18010). The tree was created using MEGA ver. 10.1.7 (available at https://www.megasoftware.net, Stecher *et al.* 2020).

**Figure S2.** The bifurcated carpel in *bip* mutant. The exposed ovule is outlined in red in the mutant.

**Figure S3.** Amplification of *WOX3* genes in wild type and *bip*. 903-bp and 807-bp fragments of *HvWOX3* and *NLD1*, respectively, were amplified with forward primer (5ʹ-ATCCTGGAGGAGATGTACCG-3ʹ) and reverse primer (5ʹ-GCTGCTCCTCCTTGATCG-3ʹ) for *HvWOX3*, and forward primer (5ʹ-AGCAGCTGATGATCCTGGAG-3ʹ) and reverse primer (5ʹ-AGGTGGAGCAAGAGGAGGAC-3ʹ) for *NLD1* using wild-type (left lane) and *bip* (right lane) genomic DNA. Arrow heads indicate the target bands.

**Figure S4.** Leaf-blade phenotypes of wild type and *bip* mutants. (A) A close-up of the lamina-joint of the second leaf in wild type and *bip*. (B–E) Epidermal cells (B, C) and cross-sections (D, E) of leaf margins of the second leaf blade in wild type (B, D) and *bip* (C, E). Scale bars = 100 μm (B–E).

**Figure S5.** Expression patterns of the *HvWOX3* and *NLD1* genes in wild type. (A–C) Double-target *in situ* hybridization of *HvWOX3* and *NLD1* genes in the central part of leaf primordium in wild-type plants. A cross-sections of P3 leaf primordium at the second-leaf stage was hybridized with the anti-sense probes of *HvWOX3* (A) and *NLD1* (B). Merged view of (A) with (B) is shown in (C). Scale bars = 50 μm.

**Figure S6.** A cross-section of spikelets in wild type hybridized with *HvWOX3* sense probe.

plac019_suppl_Supplementary_Material

plac019_suppl_Supplementary_Data

## Data Availability

All data used for analyses are provided in the accompanying Excel file as **Supporting Information**.

## Notes

**Phenome, Genome & Environment. Chief Editor:** Colleen Doherty
