# Supplementary material for: A bifurcated palea mutant infers functional differentiation of WOX3 genes in flower and leaf morphogenesis of barley
Source: AoB Plants. 2022 May 5;14(3):plac019. doi: 10.1093/aobpla/plac019 (PMC9162124; doi:10.1093/aobpla/plac019)
Supplement: plac019_suppl_Supplementary_Material [file plac019_suppl_supplementary_material.pdf]

Table S1 PCR primers used for genetic mapping of *BIP*.

Primers	Forward(5'-3')	Reverse(5'-3')	Marker type	Amplicon size(bp)	Restriction enzyme	Diagnostic band *pa1* (bp)	Diagnostic band OUI026 (bp)
AL504086-ML/-R	GACTTCGAGCTCCTCCACAC	AAAGCTTGAGCCTTCGATGA	CAPS	ca 500	*Spe*I	ca 350	ca 500
k07810	Sato et al. (2009)	Sato et al. (2009)	CAPS	ca 350	*Hap*II	ca 350	ca 200
k01252GR	Sato et al. (2009)	Sato et al. (2009)	CAPS	298	*Hha*I	188	298
WOX-like	CGCAGATCCAGCAGATCAC	GTAGTAGTGCGCGCAGGAGA	Dominant	158	no	null	158
k07273	Sato et al. (2009)	Sato et al. (2009)	Dominant	ca 1000	no	null	1000
AV917225	TTGATCAAGAGAACGCATTG	AGCTGCATCGGCAACTTC	Dominant	200	no	null	200
k08590	Sato et al. (2009)	Sato et al. (2009)	CAPS	ca 350	*Pst*I	ca 350	ca 250
k00321GR	Sato et al. (2009)	Sato et al. (2009)	CAPS	1250	*Xsp*I	271	380
k07686GR	TGGTTGCATCCCTAAGCATT	TTGGGATCGGTAAGAATCTGCTCACCAT	dCAPS	223	*Nla*III	198	230
k08747GR	Sato et al. (2009)	Sato et al. (2009)	CAPS	343	Hpy8I	200	234
k00894GR	TGGTGTTTTACATTTCGGCA	ACCAAGAAGAACGGCTCGGACCTGATCGCC	dCAPS	132	*Hpa*II	100	132
k04911	Sato et al. (2009)	Sato et al. (2009)	CAPS	ca 500	*Rsa*I	ca 380	ca 370

Markers with postscript GW are originally developed in this study by sequencing the parents.

WOX-like was designed on the basis of CAJX010219123.

Table S2 Chromosomal deletion analysis surrounding the *BIP* locus using primers that were designed on the bases of the version of barley genome assembly Hv_IBSC_PGSB_v2 of cv. Morex.

Taq polymerase Used		Taq polymerase Used						
KOD Plus Neo		QuickTaq		Barley gene	Forward primer	Reverse primer	Amplicon size	Position
KN29(WT)	*bip* mutant	KN29(WT)	*bip* mutant					
+	+	−	−	HORVU1Hr1G010190	AATGGCCAGCATGTGCAATG	ACATGCTCAACATCCAACCCA	596 bp	
***	***	+	+	HORVU1Hr1G010210	CACCCATGGGCCTTTACCTT	GCTAGCTAGCCGGTGAGATG	527 bp	
***	***	−	−	HORVU1Hr1G010230	CTGTTCCTCACTGGTCGGTC	ACACACCGACGAACAGAACA	563 bp	
+	+	−	−	HORVU1Hr1G010250	CCCTCGAGTATCATGTGGCC	GTAAGCTCGCTGGGAAGTGT	520 bp	
+	+	−	−	HORVU1Hr1G010260	GCTGTGCAAGTTGATGCCAA	TTGCCCCCTAGAGCCCTAAT	569 bp	
+	−	+	+	HORVU1Hr1G010300	TCACGCAAAAACACGTCGAC	CGCTTCACGGTTTGTCTTGG	578 bp	
+	−	+	+	HORVU1Hr1G010320	GGCTATCAAGTGTCATCACCCA	TCAGCATCGAATTACCCTGGT	567 bp	
***	***	+	+	HORVU1Hr1G010330	AATTTCCTGTGCAAGCAACTT	ATGTTGGATCAGGCCTGCAA	515 bp	
+	−	+	−	HORVU1Hr1G010360	GCAGAACTCGCATCAAGCAG	ACTGAGCTATCAATCGGGACC	597 bp	chr1H:23596766-23599186
+	−	+	−	HORVU1Hr1G010370	GCATGGTACCAGTACTCCCG	GTTTTAGGACCTGGCGGACA	566 bp	
+	−	+	−	HORVU1Hr1G010380	AGAGAACGCATTGCAGAGCT	CTTCTGCGGTTTCGTTGTCG	538 bp	
+	−	+	−	HORVU1Hr1G010390	CTGGTGCTGCAGACTGAGAA	GCAGTCTGCGATGAGACCAT	554 bp	
−	−	+	−	HORVU1Hr1G010490	CTCATCTCAGCTCGGTCACC	GGTGGAACGGAGGACTTACG	589 bp	
+	−	+	−	HORVU1Hr1G010530	CGATAGCACAACAAGACCCCT	CAGGAACGCGTTGTAGACCA	530 bp	
***	***	+	−	HORVU1Hr1G010600	CATCCTCCTTGCCCTCTTGG	CGGAAACACAAACACGTCCC	546 bp	
−	−	+	−	HORVU1Hr1G010670	TAGAGCTGAAGAACGCGACC	ACTACTACGCGCACACACAA	562 bp	
−	−	+	−	HORVU1Hr1G010730	TTGGCATCGACATGGAGACG	CATTTTAGCTCCGGGCATGC	576 bp	
+	−	+	−	HORVU1Hr1G010750	AGGGTGTTGCCAAGTCAGTT	CCAACTAATGCCGGTCTCGA	356 bp	chr1H:24839121-24842597
+	+	+	+	HORVU1Hr1G010780	GGGAAATGGGTACTCTGCCC	CAATGGTTTGCTGCCGGAAA	596 bp	
+	+	+	+	HORVU1Hr1G010830	AAATGCATGGCGATGGATGC	CAACACAGACACGATTGCGG	591 bp	
+	+	+	+	HORVU1Hr1G010890	CTCAAGGCCCATGACCTCAG	CTAGGTCAGCTCGGCACAAA	531 bp	
+	+	+	+	HORVU1Hr1G010950	ATAAGGTGGACGTGGTGCTG	TAGCCTGCGAAAAGTCAGCA	518 bp	
+	+	+	+	HORVU1Hr1G011030	CCCCCGATGAAGCCAAAGAT	AGCAGCAGAGGCCAAGAAAT	560 bp	

Notes:

(yellow)	Chromosomal deletion sites
+	Amplified
−	Not amplified
−	Not tested
***	Uncertain results (low reproducibility)

Table S3 The putative *cis*-acting regulatory elements commonly included in the promoter of *WOX3* genes in barley and rice.

Component Name	Organism	Position in (+) strand	Sequence	Function
ABRE	*Arabidopsis thaliana*	-1835	ACGTG	*cis*-acting element involved in the abscisic acid responsiveness
	Arabidopsis thaliana	-1520	ACGTG	*cis*-acting element involved in the abscisic acid responsiveness
	Hordeum vulgare	-1227	CGTACGTGCA	*cis*-acting element involved in the abscisic acid responsiveness
	Arabidopsis thaliana	-1224	ACGTG	*cis*-acting element involved in the abscisic acid responsiveness
	Arabidopsis thaliana	-1194	ACGTG	*cis*-acting element involved in the abscisic acid responsiveness
	Arabidopsis thaliana	-745	ACGTG	*cis*-acting element involved in the abscisic acid responsiveness
	Arabidopsis thaliana	-716	ACGTG	*cis*-acting element involved in the abscisic acid responsiveness
ARE	*Zea mays*	-274	AAACCA	*cis*-acting regulatory element essential for the anaerobic induction
AT~TATA-box	*Arabidopsis thaliana*	-1300	TATATA	
	Arabidopsis thaliana	-1132	TATATA	
	Arabidopsis thaliana	-1033	TATATA	
	Arabidopsis thaliana	-922	TATATA	
	Arabidopsis thaliana	-805	TATATA	
	Arabidopsis thaliana	-741	TATATA	
Box 4	*Petroselinum crispum*	-900	ATTAAT	part of a conserved DNA module involved in light responsiveness
CAAT-box	*Nicotiana glutinosa*	-2259	CAAT	
	Nicotiana glutinosa	-2201	CAAT	
	Pisum sativum	-2197	CAAAT	common *cis*-acting element in promoter and enhancer regions
	Nicotiana glutinosa	-2166	CAAT	
	Pisum sativum	-2154	CAAAT	common *cis*-acting element in promoter and enhancer regions
	Arabidopsis thaliana	-2119	CCAAT	common *cis*-acting element in promoter and enhancer regions
	Nicotiana glutinosa	-2118	CAAT	
	Pisum sativum	-2054	CAAAT	common *cis*-acting element in promoter and enhancer regions
	Pisum sativum	-2043	CAAAT	common *cis*-acting element in promoter and enhancer regions
	Pisum sativum	-2031	CAAAT	common *cis*-acting element in promoter and enhancer regions
	Nicotiana glutinosa	-1942	CAAT	
	Nicotiana glutinosa	-1602	CAAT	
	Nicotiana glutinosa	-1597	CAAT	
	Arabidopsis thaliana	-1553	CCAAT	common *cis*-acting element in promoter and enhancer regions
	Nicotiana glutinosa	-1552	CAAT	
	Pisum sativum	-1537	CAAAT	common *cis*-acting element in promoter and enhancer regions
	Arabidopsis thaliana	-1471	CCCAATTT	common *cis*-acting element in promoter and enhancer regions
	Arabidopsis thaliana	-1470	CCAAT	common *cis*-acting element in promoter and enhancer regions
	Nicotiana glutinosa	-1469	CAAT	
	Nicotiana glutinosa	-1251	CAAT	
	Nicotiana glutinosa	-1152	CAAT	
	Nicotiana glutinosa	-843	CAAT	
	Arabidopsis thaliana	-800	CCAAT	common *cis*-acting element in promoter and enhancer regions
	Pisum sativum	-774	CAAAT	common *cis*-acting element in promoter and enhancer regions
	Arabidopsis thaliana	-666	CCAAT	common *cis*-acting element in promoter and enhancer regions
	Nicotiana glutinosa	-665	CAAT	
	Nicotiana glutinosa	-629	CAAT	
	Nicotiana glutinosa	-617	CAAT	
	Nicotiana glutinosa	-426	CAAT	
CGTCA-motif	*Hordeum vulgare*	-1833	CGTCA	*cis*-acting regulatory element involved in the MeJA-responsiveness
	Hordeum vulgare	-1820	CGTCA	*cis*-acting regulatory element involved in the MeJA-responsiveness
	Hordeum vulgare	-98	CGTCA	*cis*-acting regulatory element involved in the MeJA-responsiveness
	Hordeum vulgare	-41	CGTCA	*cis*-acting regulatory element involved in the MeJA-responsiveness
G-box	*Zea mays*	-1835	CACGTC	*cis*-acting regulatory element involved in light responsiveness
	Zea mays	-1815	CACGAC	*cis*-acting regulatory element involved in light responsiveness
	Arabidopsis thaliana	-1520	TACGTG	*cis*-acting regulatory element involved in light responsiveness
	Arabidopsis thaliana	-1225	TACGTG	*cis*-acting regulatory element involved in light responsiveness
	Arabidopsis thaliana	-1194	TACGTG	*cis*-acting regulatory element involved in light responsiveness
	Arabidopsis thaliana	-745	TACGTG	*cis*-acting regulatory element involved in light responsiveness
	Arabidopsis thaliana	-717	TACGTG	*cis*-acting regulatory element involved in light responsiveness
MYB	*Arabidopsis thaliana*	-2122	CAACCA	
	Arabidopsis thaliana	-1494	CAACCA	
	Arabidopsis thaliana	-257	CAACCA	
MYC	*Arabidopsis thaliana*	-2250	CATGTG	
	Arabidopsis thaliana	-2154	CATTTG	
	Arabidopsis thaliana	-2043	CATTTG	
	Arabidopsis thaliana	-1262	CATGTG	
STRE	*Arabidopsis thaliana*	-2000	AGGGG	
	Arabidopsis thaliana	-1625	AGGGG	
	Arabidopsis thaliana	-1526	AGGGG	
TGACG-motif	*Hordeum vulgare*	-1833	TGACG	*cis*-acting regulatory element involved in the MeJA-responsiveness
	Hordeum vulgare	-1820	TGACG	*cis*-acting regulatory element involved in the MeJA-responsiveness
	Hordeum vulgare	-98	TGACG	*cis*-acting regulatory element involved in the MeJA-responsiveness
	Hordeum vulgare	-41	TGACG	*cis*-acting regulatory element involved in the MeJA-responsiveness
WRE3	*Pisum sativum*	-2413	CCACCT	
	Pisum sativum	-1932	CCACCT	
as-1	*Arabidopsis thaliana*	-1833	TGACG	
	Arabidopsis thaliana	-1820	TGACG	
	Arabidopsis thaliana	-98	TGACG	
	Arabidopsis thaliana	-41	TGACG	

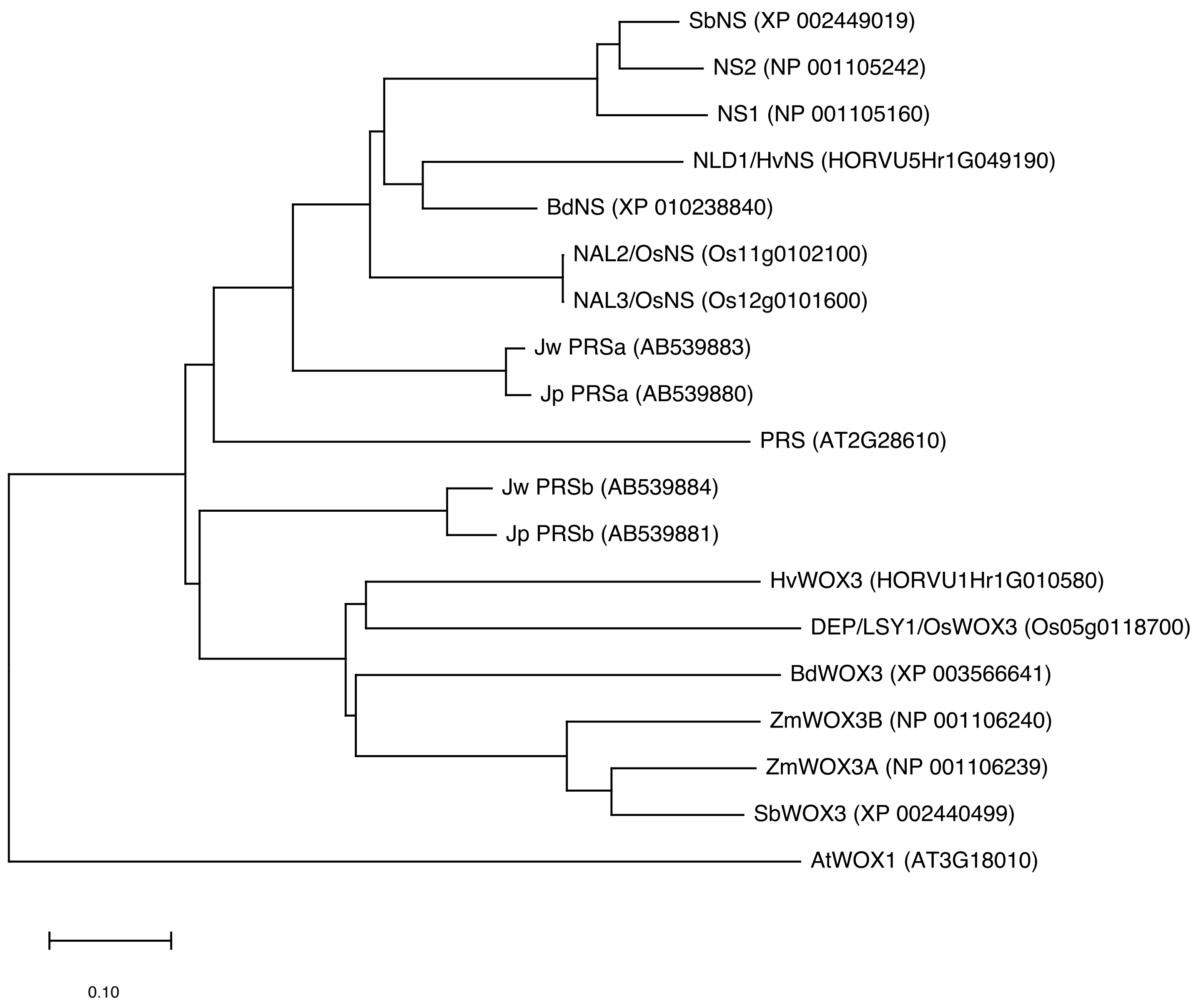

Figure S1 Phylogenetic tree of WOX3-related proteins.
WOX3-related proteins in barley (NLD1/HvNS; HORVU5Hr1G049190, HvWOX3; HORVU1Hr1G010580), maize (NS1; NP_001105160, NS2; NP_001105242, ZmWOX3A; NP_001106239, ZmWOX3B; NP_001106240), rice (NAL2; LOC_Os11g01130, NAL3; LOC_Os12g01120, DEP/LSY1/OsWOX3; LOC_Os05g02730), sorghum (SbNS; XP_002449019, SbWOX3; XP_002440499), *Brachypodium distachyon* (BdNS; XP_010238840, BdWOX3; XP_003566641), *Juncus prismatocarpus* (Jp PRSa; AB539880, Jp PRSb; AB539881), *Juncus wallichianus* (Jw PRSa; AB539883, Jw PRSb; AB539884), and *Arabidopsis thaliana* (PRS; AT2G28610, AtWOX1; AT3G18010). The tree was created using MEGA ver. 10.1.7 (available at, https://www.megasoftware.net, Stecher et al., 2020).

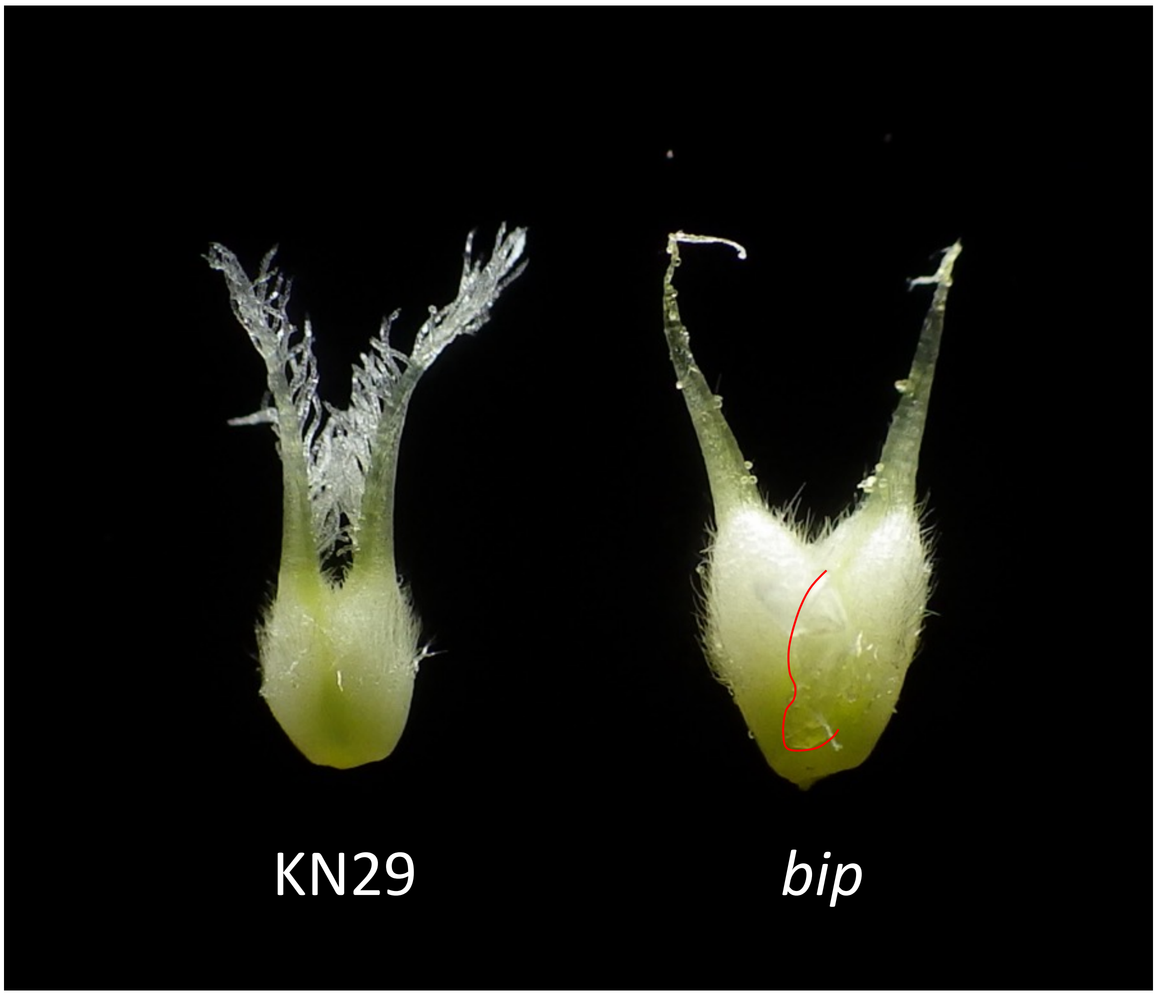

Figure S2 The bi-furcated pistil in *bip* mutant.
The exposed ovule is outlined in red in the mutant.

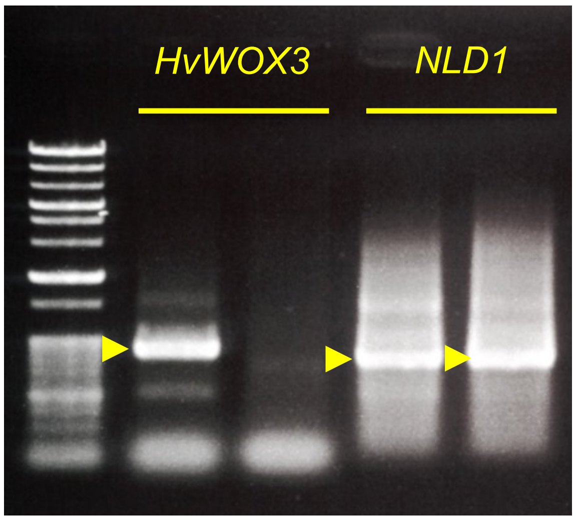

Figure S3 Amplification of *WOX3* genes in wild-type and *bip*.
903bp and 807bp fragments of *HvWOX3* and *NLD1*, respectively, were amplified with forward primer (5'-ATCCTGGAGGAGATGTACCG-3') and reverse primer (5'-GCTGCTCCTCCTTGATCG-3') for *HvWOX3*, and forward primer (5'-AGCAGCTGATGATCCTGGAG-3') and reverse primer (5'-AGGTGGAGCAAGAGGAGGAC-3') for *NLD1* using wild-type (left lane) and *bip* (right lane) genomic DNA. Arrow heads indicate the target bands.

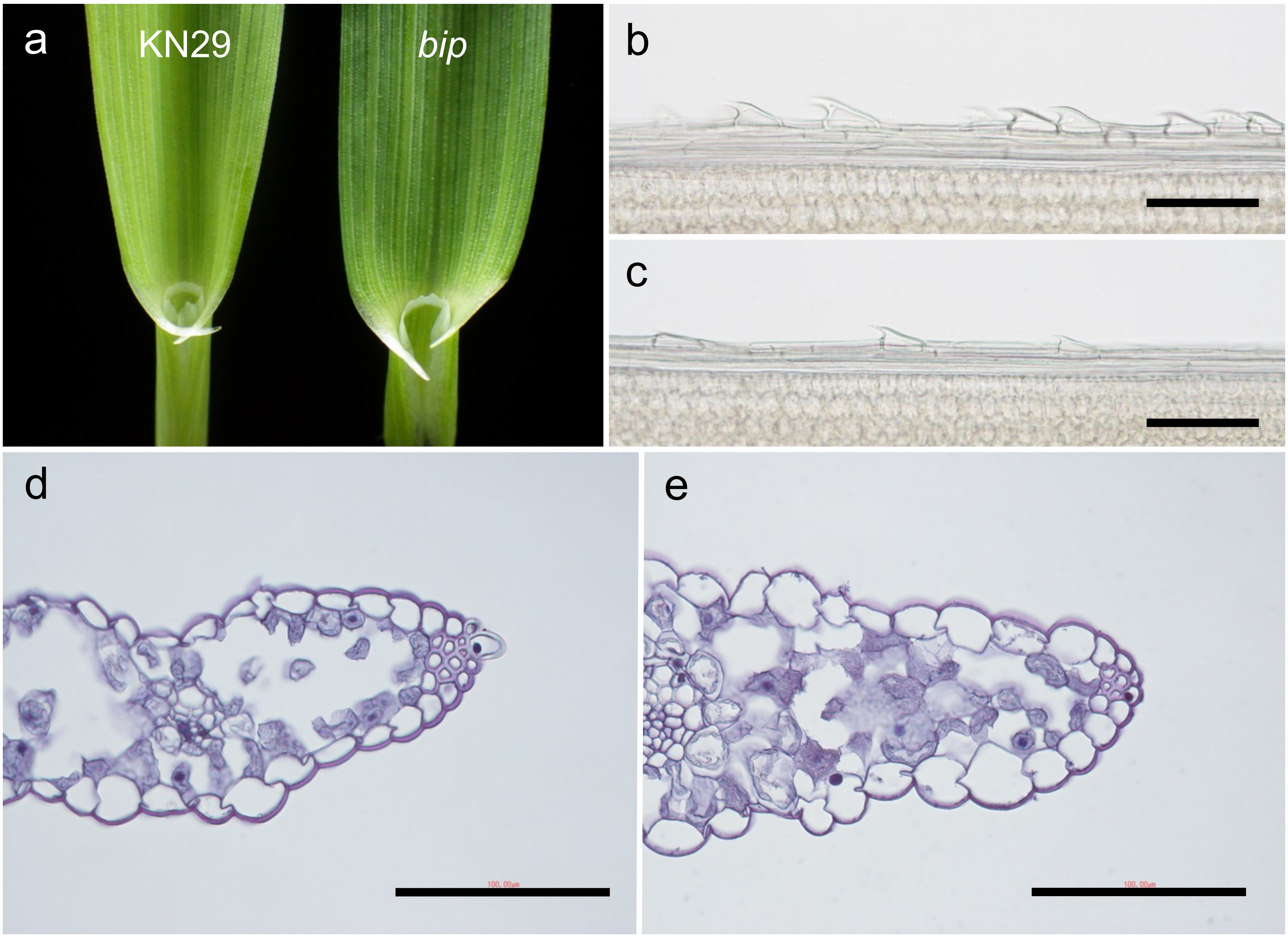

Figure S4 Leaf-blade phenotypes of wild-type and *bip* mutants.
(a) A close-up of the lamina-joint of the second leaf in wild-type and *bip*. (b-e) Epidermal cells (b,c) and cross sections (d,e) of leaf margins of the second leaf-blade in wild-type (b,d) and *bip* (c,e). Scale bars = 100μm (b-e).

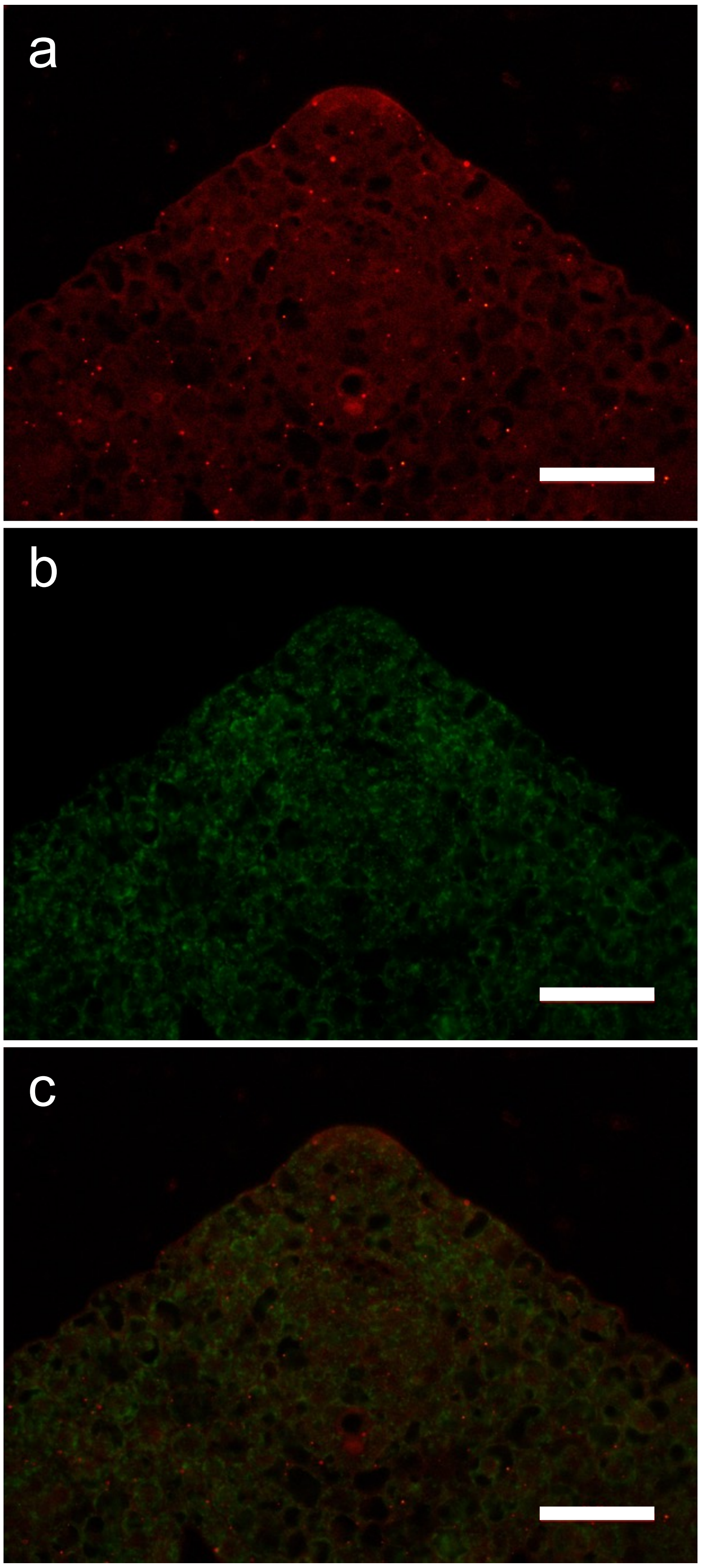

Figure S5 Expression patterns of the *HvWOX3* and *NLD1* genes in wild-type.
(a-c) Double-target *in situ* hybridization of *HvWOX3* and *NLD1* genes in the central part of leaf primordium in wild-type plants. A cross sections of P3 leaf primordium at the second-leaf stage was hybridized with the anti-sense probes of *HvWOX3* (a) and *NLD1* (b). Merged view of (a) with (b) is shown in (c). Scale bars = 50μm.

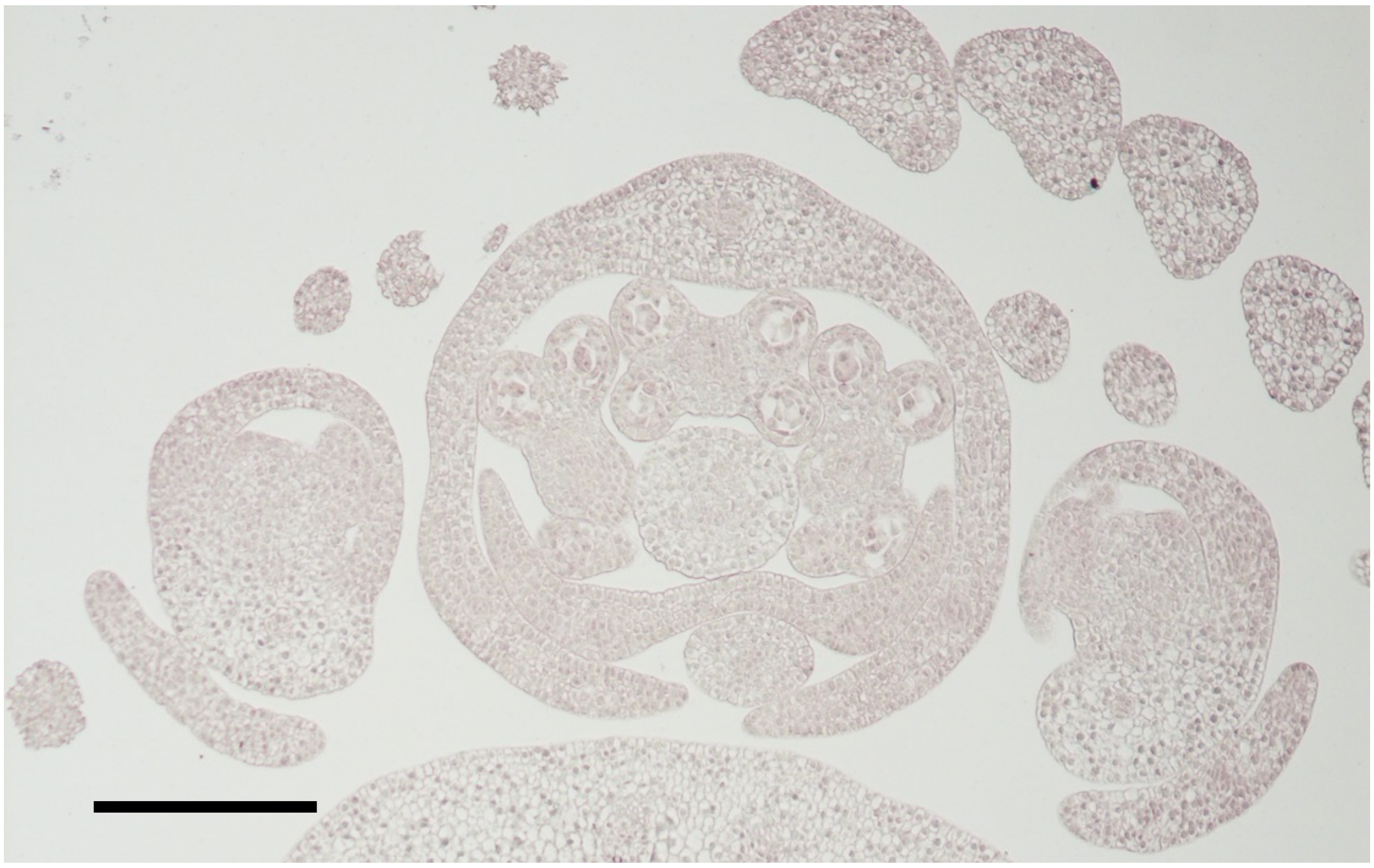

Figure S6 A cross section of spikelets in wild-type hybridized with *HvWOX3* sense probe.
